# Home-Based Electrochemical Rapid Sensor (HERS): A Diagnostic Tool for Bacterial Vaginosis

**DOI:** 10.3390/s23041891

**Published:** 2023-02-08

**Authors:** Melissa Banks, Farbod Amirghasemi, Evelyn Mitchell, Maral P. S. Mousavi

**Affiliations:** 1 Alfred E. Mann Department of Biomedical Engineering, Viterbi School of Engineering, University of Southern California, Los Angeles, CA 90033, USA; 2 Keck School of Medicine, University of Southern California, Los Angeles, CA 90033, USA

**Keywords:** bacterial vaginosis, trimethylamine, potentiometry, female reproductive health, point-of-care sensor, thread-based sensor

## Abstract

Bacterial vaginosis (BV) is the most frequently occurring vaginal infection worldwide, yet it remains significantly underdiagnosed as a majority of patients are asymptomatic. Untreated BV poses a serious threat as it increases one’s risk of STI acquisition, pregnancy complications, and infertility. We aim to minimize these risks by creating a low-cost disposable sensor for at-home BV diagnosis. A clinical diagnosis of BV is most commonly made according to the Amsel criteria. In this method, a fish-like odor, caused by increased levels of trimethylamine (TMA) in vaginal fluid, is used as a key diagnostic. This paper outlines the development of a Home-Based Electrochemical Rapid Sensor (HERS), capable of detecting TMA in simulated vaginal fluid (sVF). Instead of odor-based detection of volatilized TMA, we identify TMA in trimethylammonium form by utilizing HERS and a potentiometric readout. We fabricated the ion selective electrode using a carbon-black-coated cotton string and a TMA-selective membrane consisting of calix[4]arene and sodium tetrakis[3,5-bis(trifluoromethyl)phenyl]borate. When paired with a standard reference electrode, our device was able to quantify TMA concentration in deionized (DI) water, as well as sVF samples at multiple pH levels with a clinically relevant limit of detection (8.66 µM, and theoretically expected Nernstian slope of 55.14 mV/decade).

## 1. Introduction

**Figure 1:**
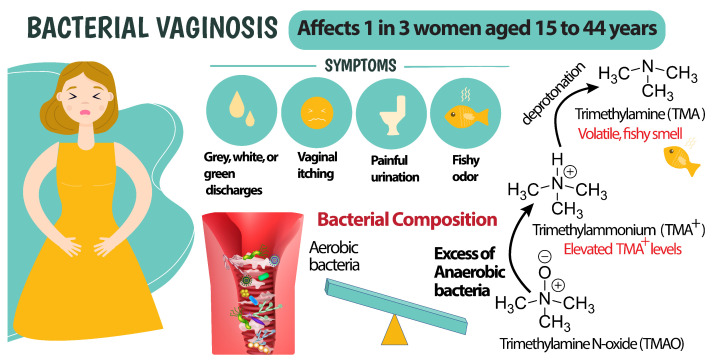
Bacterial vaginosis symptoms and TMA production mechanism. A reduction in TMAO caused by anaerobic bacteria results in elevation of TMA levels in vaginal discharge.

Despite recent advances in point-of-care and at-home sensing technologies, and wearables, very little attention has been given to developing new tools for medical conditions that are specific to women. Vaginal symptoms are some of the most common complaints amongst women seeking gynecological care [1]. Bacterial vaginosis is the most commonly occurring vaginal infection worldwide, affecting between 29 and 33% of reproductive-aged women [2], and has a 50% recurrence rate within a year’s time [3]. Despite its prevalence, BV has historically been significantly underdiagnosed, as 84% of those infected remain asymptomatic [4]. Figure 1 outlines the commonly reported BV symptoms including (1) thin white or gray vaginal discharge, (2) pain, itching, or burning in the vagina, (3) painful urination, and (4) a strong fish-like odor, especially after sex [5].

If left untreated, bacterial vaginosis compromises the protective acid mantle of the vagina, leaving individuals at greater risk of contracting STIs such as HIV [6], trichomonas [7], herpes simplex virus type 2 [8], gonorrhea, chlamydia [9], and pelvic inflammatory disease [10]. Pregnancy complications, preterm birth [11], and postabortal infection [12] have also been tied to BV. Given that both African-American and Mexican-American women are disproportionately affected by the infection, BV is thought to be a contributing factor to the racial disparity present in STI acquisition and frequency of preterm birth [4]. The direct medical costs of BV treatment result in a USD 1.3 billion annual expenditure by the US healthcare system, not including an additional USD 1.76 billion spent on BV-associated preterm births and HIV infection [2].

A clinical diagnosis of BV is made according to one of two standards, the Nugent criteria and the Amsel criteria. Although the Nugent criteria is generally considered to be the gold standard for diagnosis, it is costly in terms of time and resources as it requires vaginal swab sample examination by a microscopist for levels of Gram-positive and Gram-variable rods. The additional time, equipment, and trained personnel needed to use the Nugent criteria make its use far less common in the clinical setting [13]. Alternatively, the Amsel criteria stipulates the demonstration of three out of four BV diagnostic criteria: (1) homogeneous, thin discharge (milk-like consistency) that smoothly coats the vaginal walls, (2) clue cells (e.g., vaginal epithelial cells studded with adherent bacteria) upon microscopic examination, (3) pH of vaginal fluid > 4.5, and (4) a fishy odor of vaginal discharge before or after the addition of 10% KOH (i.e., the whiff test) [11].

**Figure 2:**
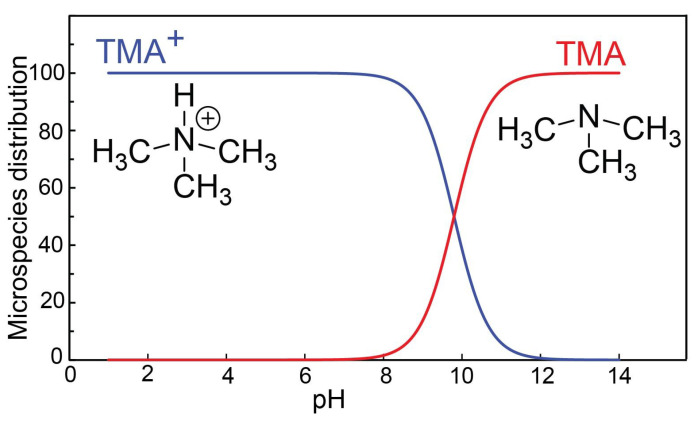
Distribution of TMA and TMA${}^{+}$ at different pH values. Distribution is calculated using a pKa value of 9.8 for TMA.

The fish-like odor referred to in Figure 1 is caused by elevated levels of vaginal trimethylamine. The increasing numbers of anaerobic bacteria within the vaginal microbiome reduce the trimethylamine N-oxide, and create elevated TMA levels [14]. As the ratio of anaerobic:aerobic bacteria increases in those affected with BV, TMA concentration and vaginal pH rise as a result [15]. TMA is a strong base (pKa of trimethylammonium 9.8 [16]). At pH values lower than 8, more than 97% of TMA is present in the protonated trimethylammonium form, and has a positive charge. Figure 2 shows the distribution of protonated and unprotonated TMA at different pH values. TMA in the unprotonated form (amine form) is a volatile organic compound, with an associated fishy smell. The formation of TMA and the deprotonation of TMA${}^{+}$ is utilized in the Amsel criteria, where 10% KOH is added to the vaginal discharge, and the fishy smell is characterized as a positive criterion for the diagnosis of BV [11]. Known colloquially as the "whiff test", the physician smells the sample to see if the distinctive fishy odor is produced. While this method is certainly efficient in terms of time and cost, it is relatively subjective due to its dependence upon the olfactory prowess of individual healthcare providers.

Surprisingly, there are very few point-of-care (POC) technologies for the diagnosis of BV. In the realm of at-home diagnostics, many products such as the Vagisil Screening Kit for Vaginal Infections, FloriSense Women’s Vaginal Health Test, and CVS Health Feminine Screening Kit for Vaginal Infection exist. These kits only measure vaginal pH. This measurement can be useful in predicting vaginitis (elevated pH is a symptom) and potentially ruling out a yeast infection. However, pH measurement alone does not provide information on the cause of elevated pH, which is a common symptom in both BV and trichomoniasis [17,18]. Additionally, kits based on pH measurement cannot be used during menstruation when pH levels are impacted by the presence of blood [19], nor will they be accurate after sexual intercourse [20]. Other manufacturers such as myLAB Box and Walk-In Lab allow users to collect their own vaginal fluid sample at home and mail it in for microscopic analysis based on NC. Both products, however, require 6–8 days to produce results and are vastly more expensive than other over the counter options (USD 189 and USD 175, respectively) [17]. Clinical POC options include OSOM BVBlue which detects sialidase, a bacterial byproduct, within vaginal fluid. Although the test is rapid (<10 min), it is often prohibitively expensive and it cannot rule out the presence of other infectious agents such as C. Albicans and T. Vaginalis, nor can it be used on women who have recently engaged in sexual intercourse [21]. HERS avoids the storage limitations and stability issues of immunosensors, as well as the time-intensive process of aptamer development and isolation [22]. We believe these qualities will allow HERS to stand out as a low-cost option able to be easily shipped and stored by healthcare facilities, at home or abroad.

Very few academic works have focused on point-of-care or at-home devices for the diagnosis of BV. Pal et al. repurposed a wearable sweat patch for measuring vaginal pH [23]. Liu et al. developed a field effect transistor based on carbon nanotubes for the measurement of pH and biogenic amines (putrescine and cadaverine) [24]). Pawley et al. developed a paper-based assay for the detection of clue cells in vaginal fluid as a diagnostic measure for BV [25]. This work is the first to conceptualize a TMA${}^{+}$ sensor, and to measure TMA${}^{+}$ (instead of using the TMA smell test) for the diagnosis of BV. This study has several layers of innovation. Firstly, we are the first to develop a yarn-based sensor for the diagnosis of BV. Traditional lab-on-a-chip devices are rigid, whereas our yarn-based sensor has the advantages of a traditional lab-on-a-chip but with the flexibility, comfort, and low cost of a textile-based device. This work utilizes a calix[4]arene ionophore for developing a TMA${}^{+}$ potentiometric sensor for the first time, and characterizes the properties of this new sensing membrane. This work is the first report of a yarn-based electrochemical TMA${}^{+}$ sensor. It is our hope that the HERS platform can pave the way for a more inclusive healthcare system, and more efficient tools for diagnosis and preventative care for gynecological health.

## 2. Materials and Methods

### 2.1. Materials and Reagents

Sodium tetrakis[3,5-bis(trifluoromethyl)phenyl]borate (NaTPB), calix[4]arene (CX4), 2-nitrophenyl octyl ether (o-NPOE), high-molecular-weight poly(vinyl chloride) (PVC), tetrahydrofuran (THF, inhibitor-free, for HPLC), Trimethylammonium chloride (TMA), potassium hydrogen phosphate, potassium dihydrogen phosphate, D-(+)-Glucose monohydrate, potassium chloride, sodium chloride, calcium chloride, sodium hydroxide, hydrochloric acid (HCl), and tetrabutylammonium bromide (Bu${}_{4}N^{+}$) were purchased from Sigma-Aldrich. The encapsulating ink Sally Hansen Rapid Dry Nail Polish was purchased from Amazon.com. Commercial vaginal fluid simulant (Conceive Plus Fertility Lubricant) was purchased from Conceive Plus via Amazon.com, item number: CP-00001, ASIN: B002V0ZJ4Y. Ingredients of Conceive Plus Fertility Lubricant include water, hypromellose, sodium chloride, methylparaben, glycerol, sodium phosphate, potassium chloride, sodium dihydrogen phosphate, magnesium chloride, and calcium chloride. Deionized water (conductivity of 18.20 MΩ/cm) was used to prepare all solutions.

### 2.2. Sensor Fabrication

**Figure 3:**
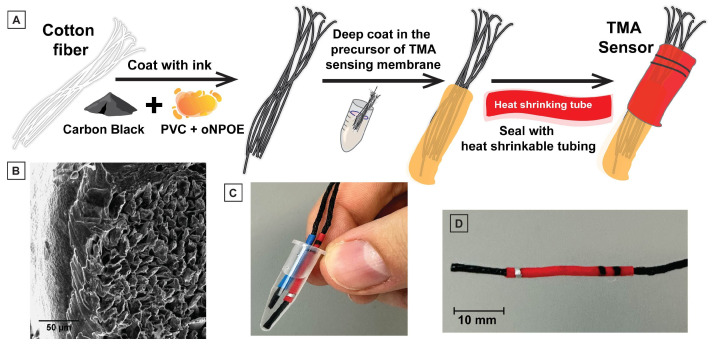
(**A**) The steps for fabrication of the HERS. (**B**) The SEM image of the cross section of carbon-ink- and membrane-coated TMA sensor (scale bar: 50 µm). (**C**,**D**) Photographs of the HERS platform.

Figure 3 demonstrates the manner in which we fabricated the HERS. Conductive ink was prepared by mixing 250 mg of PVC and 500 mg of o-NPOE in 3 mL THF (per gram of mixture) for 3 h. Then, carbon black powder was crushed into the polymeric matrix using a mortar and pestle. A 6-cm-long 100% cotton fiber was coated with the conductive ink containing 25 wt% carbon black powder with an average conductivity of 300 Ω/cm, allowing it to dry overnight. The TMA selective precursor was made by mixing 330 mg of PVC, 660 mg of o-NPOE, 10 mg of NaTPB, and 5.7 mg of CX4. The TMA selective precursor was dissolved in 3 mL THF per gram of mixture. The precursor was stirred overnight until it became homogeneous and transparent. Next, we added 18 µL of 1M TMA to make the precursor selective toward TMA and it was stirred overnight, where an opaque precursor formed. We fabricated all-solid-state TMA selective electrodes by dipping a conductive fiber in a TMA selective precursor. The last 1 cm at the ends of the coated yarn electrodes was dipped for 10 s into the TMA selective precursor three times and was allowed to dry for 10 s. The electrodes were allowed to dry overnight. We applied an encapsulating ink (commercially available nail polish purchased from amazon.com) to the middle section of the electrodes, leaving 1 cm of membrane and 1 cm of the uncoated electrode exposed on either end. Next, we covered the nail-polished region with a commercially available heat-shrinking tube to create a hydrophobic barrier.

### 2.3. Measurement Protocols

We performed the potentiometric measurements using an EMF 16-channel potentiometer (Lawson Labs, Malvern, PA, USA) controlled via EMF Suite 2.0 software (Lawson Labs). We used an Ag/AgCl double-junction-type external reference electrode (Mettler Toledo DX200, CA, USA) with 3.0 M KCl saturated with AgCl as the reference solution (inner filling solution) and 1.0 M lithium acetate as a bridge electrolyte. We used the Thermo Scientific Orion Star A211 pH kit for pH adjustments. We generated the calibration curves for TMA via stepwise dilution of solutions of TMA. The limit of detection (LOD) was determined according to IUPAC recommendations. We applied two linear regressions (electromotive force (emf) vs. log [concentration]) within two concentration ranges. The first was the region where the HERS illustrated Nernstian behavior in response to stepwise dilutions. The second was the concentration range where the emf of the TMA did not change significantly after stepwise dilutions. The intersection of these two linear regressions provided the LOD [26,27,28]. We recreated the simulated vaginal fluid based on the recipe by Tietz et al. [29]. For this purpose, we dispersed 3.50 g of sodium chloride, 10.80 g of glucose monohydrate, 1.50 g of potassium chloride, 1.74 g of potassium hydrogen-phosphate, and 1.36 g of potassium dihydrogen-phosphate in 1 L of DI water, and adjusted the pH to desired values with HCl.

## 3. Results and Discussion

**Figure 4:**
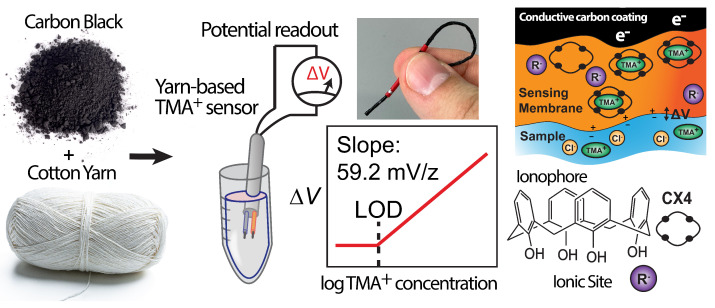
The proposed Home-Based Electrochemical Rapid Sensor, HERS. The sensor is made of carbon black and cotton yarn, and a potentiometric ion-sensing membrane. The electrochemical potential of the HERS depends linearly on the logarithm of TMA${}^{+}$ activity in the test solution. A calix[4]arene ionophore selects for TMA${}^{+}$ via complex formation in the sensing membrane. The negatively charged ionic site (R${}^{-}$) buffers the concentration of the TMA${}^{+}$ in the sensing membrane and establishes a sample-dependent potential. The charge separation of TMA${}^{+}$ and its counter anion at the interface of the sensing membrane and sample solution creates an electrical potential (emf) that is measured by the electrode.

The HERS is an electrochemical, specifically potentiometric sensor for the direct detection of TMA${}^{+}$ in vaginal fluid (VF). The detection relies upon a TMA${}^{+}$ potentiometric sensing membrane. This sensing membrane is embedded in a yarn substrate to create a portable, inexpensive, and light sensor (Figure 4). We validated the HERS in a simulated VF (sVF) and commercially acquired VF (cVF). In this work, instead of volatizing TMA using an addition of KOH, we directly detected TMA${}^{+}$ using an electrochemical ion sensor that was selective to TMA${}^{+}$. We have created a Home-Based Electrochemical Rapid Sensor (HERS) for the direct detection of TMA${}^{+}$ in vaginal discharge. As seen in Figure 4, the HERS was fabricated using cotton string and inexpensive carbon black, and enables a quantitative and accurate measure of TMA${}^{+}$ levels in vaginal fluid. The HERS can be used by the physician at the clinic, or by the patient at home. While our work is at the proof-of-concept stage, it demonstrates the feasibility of future integration into feminine hygiene products to create a fully integrated system. The HERS can be utilized by women to test for BV on a monthly basis at home, and identify both symptomatic and asymptomatic instances. The goal of this research is to reduce the BV-associated burden on our healthcare system and, more importantly, to decrease the long-term health consequences of untreated bacterial vaginosis infection.

### 3.1. Design and Fabrication of the HERS

The use and different components of the HERS are shown in Figure 4. A step-by-step fabrication of the HERS is shown in Figure 3. We utilized yarn as a substrate for sensor fabrication; yarn supports the conductive ink and the TMA${}^{+}$ sensing membrane. The low cost and light weight of yarn allows for an inexpensive fabrication process. The fabrication of the HERS is simple, and does not require microfabrication and lithography. The yarn itself defines the channel size. Yarn is available in a variety of diameters, providing options for making the HERS with different sizes. Our conductive ink is made of carbon black, which is an inexpensive high surface area material for the development of solid-contact potentiometric sensors. In prior work, we have demonstrated that we can develop stable and reproducible yarn-based potentiometric sensors for K${}^{+}$, Na${}^{+}$, and Ca${}^{2+}$ using our carbon black ink [30]. In this work, we used a similar fabrication process for developing a TMA${}^{+}$ sensor. Once the yarn was coated with the carbon black ink, and was fully dried, we coated the tip with the precursor of the TMA${}^{+}$ sensing membrane, and waited overnight for the membrane to set (forming approximately 50 µm thickness, SEM in Figure 3C). This membrane precursor contains the polymer support, plasticizer, ionophore, and ionic site (the four components of a potentiometric sensing membrane), dissolved in tetrahydrofuran. The exposed conductor was then sealed using heat-shrinkable tubing to waterproof the sensor and avoid short circuits in the measurement. A small part of the ink-coated yarn was left exposed at the other tip of the HERS to allow connection to the potentiometer for signal readout. Figure 3C,D show the resulting sensor.

### 3.2. The Sensing Mechanism in the HERS and Sensor Response

HERS is a potentiometric sensor relying on a two-electrode system for measurement. The experimental setup includes an external reference electrode and the HERS electrode, immersed in the sample solution containing TMA${}^{+}$, (see Figure 4). This work is only focused on the TMA${}^{+}$ sensor itself, and not on a yarn-based reference electrode. Therefore, measurements were taken using a commercial external reference electrode. Experiments were repeated in duplicate while the reported results are the average of five HERS electrodes per experiment. We measured the HERS electrode potential under zero-current conditions (expected in potentiometric measurements). The resultant potential is commonly known as the electromotive force (*emf*). The Nernst equation (Equation (1)) governs the correlation between the potential of *emf* and the activity of TMA ions in the sample solution [31].

(1) $$emf=E_{i}^{0}+\frac{RT}{z_{i}F}ln\left(a_{i}+\sum k_{i,j}^{pot}a_{j}^{\frac{z_{i}}{z_{j}}}\right)$$

In Equation (1), *R* is the universal gas constant, *T* is the setup temperature, *F* is the Faraday constant, *z* and *a* are the charge and activity of the primary ion, and $k_{i,j}^{pot}$ is the selectivity coefficient that quantifies the selectivity of the sensor for the primary ion *i*, over the interfering ion *j*. The theoretical value of the *RT/zF* is approximately 59.2 mV/d, considering room temperature to be 25 °C.

The TMA${}^{+}$-dependent electrical potential is generated at the interface of the TMA-sensing membrane and sample solution (see Figure 4) due to charge separation. This sensing membrane consists of a polymer matrix, which mechanically supports the components of the TMA-selective membrane. The primary components of the TMA-selective membrane are a hydrophobic ion with a charge opposite to that of the TMA${}^{+}$ ion and an ionophore that selectively binds to the TMA${}^{+}$ ion. The activity of the TMA${}^{+}$ ions in the membrane and the sample solution determine the magnitude of the generated emf [28,31].

**Figure 5:**
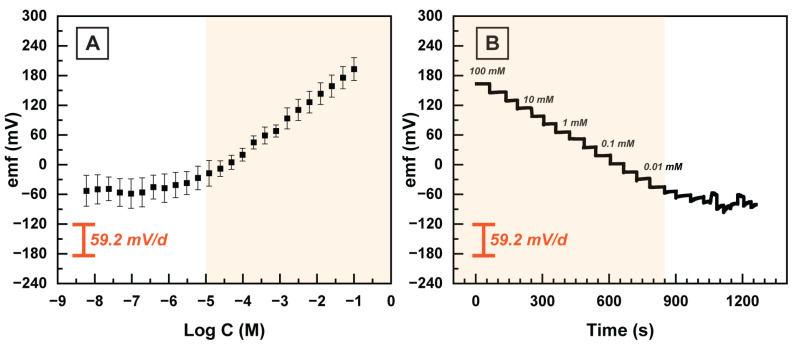
(**A**) The response of the HERS electrodes to TMA${}^{+}$ (n = 5) with TMA-selective polymeric sensing membrane. (**B**) The emf value of the HERS electrode over time during successive dilution of TMA${}^{+}$ solution. Highlighted regions correspond to the linear range, 10 µM to 100 mM.

First, we validated the sensitivity of the HERS electrode toward TMA${}^{+}$ ions by plotting the emf response of the HERS in the stepwise-diluted TMA${}^{+}$ solutions in DI water. The HERS electrodes (n = 5) illustrated a slope of 55.14 ± 4.44 mV/d (see Figure 5) comparable with the theoretical Nernstian value of 59.20 mV/d. Moreover, the LOD of the HERS electrodes is 8.66 µM, which is two orders of magnitude lower than the clinically relevant concentration of the TMA in VF [32].

### 3.3. TMA${}^{+}$ and Ionophore Interaction

One of the critical aspects of any ion-selective electrode (ISE) is its selectivity toward the target ion, in this case, TMA${}^{+}$, which permits the utilization of the electrodes in real-life applications. The energy transfer of the target ions from the sample aqueous solution to the organic polymeric membrane determines the selectivity, which depends on ion lipophilicity [33,34]. Therefore, integrating a selectively binding ionophore into the ISE sensing membrane lowers the overall free energy for ion transfer into the organic phase for those ions to which the ionophore binds [35,36]. There are commercial ionophores available for many inorganic ions such as K${}^{+}$, Na${}^{+}$, and Ca${}^{2+}$ [36].

**Figure 6:**
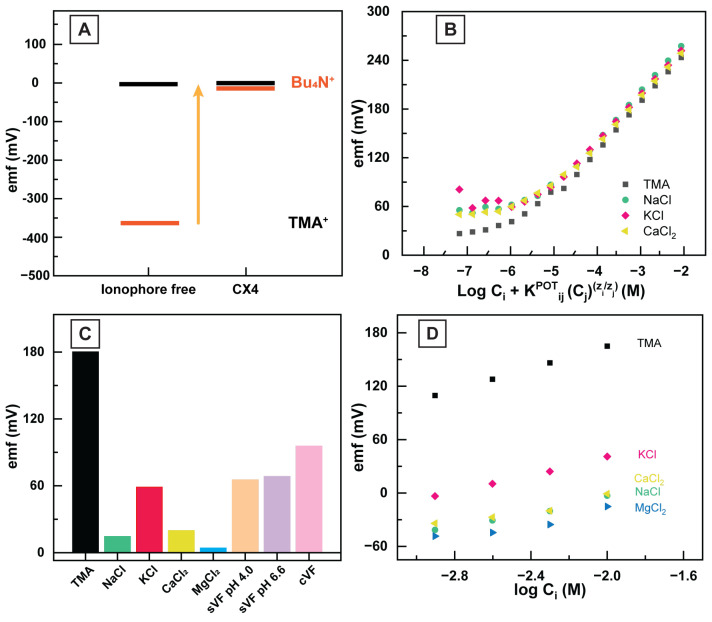
(**A**) The differences in the emf of the HERS for 0.1 mM TMA${}^{+}$ solution with respect to 0.1 mM Bu${}_{4}$N${}^{+}$ upon introducing 10.00 mg/g of CX4 to the sensing membranes. (**B**) FIM calibration: emf response of the HERS electrode in the presence of DI water, 0.1 mM NaCl, 0.1 mM KCl, and 0.1 mM CaCl${}_{2}$. (**C**) Measured emf response of the HERS in the solutions containing 10.0 mM TMA, 10.0 mM NaCl, 10.0 mM KCl, 10.0 mM CaCl${}_{2}$, 10.0 mM MgCl${}_{2}$, sVF (pH = 6.6), sVF (pH = 4.0), and cVF. (**D**) SSM calibration: emf response of the HERS electrode in various interfering ions (*j*).

There are no commercial ionophores for TMA${}^{+}$. Prior work by us and others showed that calixarenes interact strongly with quaternary ammonium ions ([28,34]). The calixarenes’ cavity sizes could be a factor in determining these receptors’ selectivity and binding strengths to target ions [34,37]. Hence, we selected CX4 as an ionophore for this study due to the TMA’s size correlation with CX4’s cavity size, and based on our prior work showing that CX4 acts as a strong ionophore for acetylcholine and choline [28,34]. To confirm the binding of TMA${}^{+}$ to CX4 and the facilitated transfer of TMA${}^{+}$ to the sensing membrane using the CX4 ionophore, we followed a protocol by Ceresa et al. [38]. This method’s reliability was previously validated by Abd El-Rahman et al. [28,34]. In this protocol, we measured the emf of the two TMA${}^{+}$ sensors, one with and one without the CX4 ionophore in the sensing membrane (experiment repeated in five replicates). We then placed these electrodes into the solution of a non-interacting reference ion (tetrabutylammonium, Bu${}_{4}$N${}^{+}$), and measured the emf of the sensors again. We chose this reference ion because its large size prevents it from binding to CX4 [28,34]. Using this reference ion also removes variabilities in electrode fabrication and creates a self-comparison for each electrode. The electrode emf values were scaled with the Bu${}_{4}$N${}^{+}$ placed as the zero reference point. As shown in Figure 6A, when CX4 is added to the sensing membrane, we see a higher emf value for TMA${}^{+}$ and a smaller emf difference between TMA${}^{+}$ and Bu${}_{4}$N${}^{+}$. Basically, in the absence of ionophore (CX4), the selectivity is determined by ion hydrophobicity only, and we observe a much higher emf for the more hydrophobic reference ion (Bu${}_{4}$N${}^{+}$) vs. TMA${}^{+}$. When the CX4 ionophore is added to the sensing membrane, it can interact with TMA${}^{+}$ in the membrane and form a complex, but there are no interactions with Bu${}_{4}$N${}^{+}$. Therefore, the emf is controlled by both the CX4-TMA${}^{+}$ interaction and ion hydrophobicity. Due to the strong complexation of CX4-TMA${}^{+}$, we see a 350 mV difference in the TMA${}^{+}$ emf between the ionophore-free membrane and the membrane with the CX4 ionophore (see Figure 6A). This large difference confirms a strong ionophore–TMA${}^{+}$ interaction in the sensing membrane.

### 3.4. Quantifying the Selectivity of HERS

We applied a well-established quantification method for the selectivity analysis of the HERS electrodes: the fixed interference method [27,35]. Here, the TMA${}^{+}$ ions and the primary ion (*i*) in the presence of an interfering ion (*j*) are conventionally quantified by the logarithm of the selectivity coefficient of a numerical quantification specifying the potential of the HERS electrode to differentiate the TMA${}^{+}$ ion from an interfering ion, which is given by Equation (2). In this equation, the terms ai and aj are the activity of TMA${}^{+}$ and the interfering ion, respectively [27].

(2) $$log\left({k_{i}}_{j}^{pot}\right)=log\left(a_{i}/a_{j}^{z_{i}/z_{j}}\right)$$

Furthermore, the separate solution methods take a different approach to quantify the selectivity of the interfering ions versus the primary ion. In this method, we measure the emf potential of the HERS electrodes within two different solutions, one containing the TMA${}^{+}$ (but no interfering ion), and the second solution containing the interfering ion (*j*) at the same concentration (but no TMA${}^{+}$), if the measured emf values for TMA${}^{+}$ and the interfering ion are $E_{i}$ and $E_{j}$, respectively. The selectivity coefficient could be calculated via Equation (3) [27]. Table 1 demonstrates the selectivity values of TMA${}^{+}$ for primary ions in sVF, including sodium, potassium, and calcium.

(3) $$log\left(\left({k_{i}}_{j}^{pot}\right)\right)=\frac{\left(E_{j}-E_{i}\right)}{RT(lna_{i})}+\left(1-\frac{z_{i}}{z_{j}}\right)\left(loga_{i}\right)$$

We investigated the feasibility of the HERS in simulated vaginal fluid to validate our proof-of-concept platform in detecting TMA${}^{+}$ in biofluid. We selected a chemically defined medium (CDM) formulation for creating sVF in this study, initially developed for studies focused on investigating specific mechanisms and factors that control microbial populations [39]. However, this recipe recapitulates the majority of the physiological ions, which might be potential interfering ions in determining TMA${}^{+}$ levels.

First, we adjusted the pH of sVF to 4.0 and 6.6, covering both healthy and unhealthy conditions [3,18]. We positioned the reference electrode and the HERS electrodes (n = 5) into the sVF and instantly started data acquisition by measuring the background emf values for 60 s; then, we spiked the sVF with 260 µM TMA${}^{+}$. We chose this level since it is the clinically relevant value of TMA${}^{+}$ in VF, as reported by Wolrath et al. as 24.5 µg TMA/gVF. We converted this concentration to µM using a VF density approximation of 1.01 g/mL [32]. We observed an immediate spike in the emf values from the baseline, confirming the selectivity of the HERS platform to TMA${}^{+}$ in the presence of interfering ions (see Figure 6).

### 3.5. Application of HERS in Vaginal Fluid

**Figure 7:**
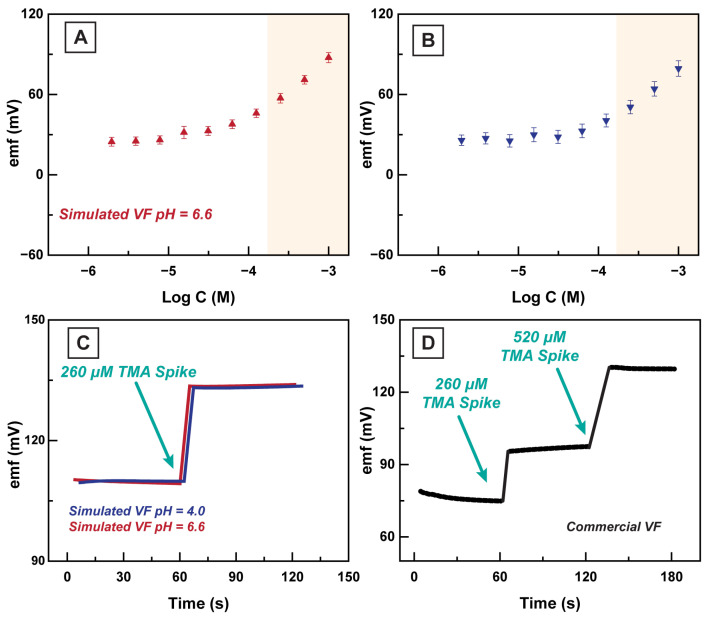
(**A**) The response of the HERS electrodes (n = 5) in sVF (pH = 6.6) to spiked TMA${}^{+}$. (**B**) The response of the HERS electrodes (n = 5) in sVF (pH = 4.0) to spiked TMA${}^{+}$. Highlighted regions correspond to the linear range of 10 µM–100 mM. (**C**) The measured emf of the HERS electrodes (n = 5) before and after spiking with 260 μM TMACl in the sVF (pH = 6.6 and pH = 4.0). (**D**) The emf trace of the HERS electrodes (n = 5) before and after spiking with 260 μM and 520 μM TMACl in the cVF.

We examined the sensitivity of the HERS in simulated vaginal fluid to validate our proof-of-concept platform in detecting TMA${}^{+}$ in the VF. We utilized the CDM formulation for creating sVF, similar to our previous experiments [19]. A commercially available fertility lubricant (Conceive Plus) was also utilized in this experiment. The purpose of this product is to relieve vaginal dryness and assist with sperm motility and viability when trying to conceive. This lubricant contains ions (Na${}^{+}$, Ca${}^{2+}$, K${}^{+}$, and Mg${}^{2+}$), and is formulated with an elevated pH (∼7) to promote sperm health [40,41]. The HERS electrodes (n = 5) and the reference electrode were placed into the sVF containing 100 µM TMA${}^{+}$ ions, which is lower than the clinically relevant value of 260 µM, to confirm the sensitivity of the HERS electrodes to TMA${}^{+}$ levels in the physiological range. Next, we performed several stepwise dilutions of 100 µM TMA${}^{+}$ in sVF background to find the sensitivity and LOD of the HERS in sVF. We observed the LOD of 52.76 µM in the sVF with an adjusted pH of 6.6, and the HERS electrodes illustrated a satisfactory sensitivity of 50.04 ± 1.49 mV/d. Moreover, we investigated the effect of pH on the performance of the HERS electrode by adjusting the pH of sVF to 4 (healthy VF pH [3,18]). In this experiment, HERS electrodes illustrated a sensitivity of 50.32 ± 2.49 mV/d with LOD being equal to 83.99 µM (see Figure 7). This experiment was repeated in cVF to validate that HERS can accurately detect TMA${}^{+}$ in this medium (see Figure 7D). A comparison chart between the HERS platform and other alternative detection methods is outlined in Table 2.

## 4. Conclusions

This work shows a proof-of-concept rapid electrochemical sensor for an at-home evaluation of BV. We fabricated a potentiometric sensor for TMA${}^{+}$ with a working range from 10 µM to 100 mM, and a response time of a few seconds by utilizing a hydrophobic calix[4]arene doped into the ion-selective membrane to improve selectivity of HERS toward TMA${}^{+}$. The HERS was successfully applied to measure TMA${}^{+}$ in simulated vaginal fluid and commercial fertility lubricant without any sample pretreatment or prior separation steps. Developing the HERS platform provides a tool for identifying TMA in vaginal fluid as a woman’s health indicator in complex matrices. By coupling the sensor with a miniaturized thread-based reference electrode, it could easily serve as a quantitative point-of-care or at-home diagnostic device for women’s health.

Despite profound health implications including increased risk of STIs, pregnancy loss, and infertility, the asymptomatic nature of many BV infections propagates its spread. In future work, we aim to integrate our TMA${}^{+}$ sensor into feminine hygiene products to enable individuals to screen for BV frequently. This frequent screening can create an early detection of potential infections, enabling seamless operation and swift treatment with antibiotics. Validation in clinical samples of healthy and BV-infected human vaginal fluid will be pursued in future work.

We also want to point out that bacterial vaginosis is a difficult infection to diagnose as it is predicated upon the delicate balance of bacteria within the vaginal microbiome. Each woman has a unique microbial constitution and baseline vaginal pH that limits the value of simulated vaginal fluid testing, requiring a personalized diagnosis. Relying on more than a single biomarker and multiplexed analysis (perhaps pH and other amines) is likely the ideal solution to achieve personalized health screening for BV and other diseases affecting female reproductive health. The compact low-cost nature of the HERS would also allow for multiple sensors (for other diagnostic criteria) to be included alongside TMA${}^{+}$ detection in the future. This would be particularly beneficial to low-resource settings where microscopy is unavailable. 

## Figures and Tables

**Table 1:** The selectivity coefficients of the HERS over potentially interfering ions (*j*).

Ions	${{\mathrm{logk}}_{i}}_{j}^{\mathrm{pot}}\left(\mathrm{SSM}\right)$	${{\mathrm{logk}}_{i}}_{j}^{\mathrm{pot}}\left(\mathrm{FIM}\right)$
Sodium	−2.69	−1.25
Potassium	−2.03	−1.12
Calcium	−3.99	−1.09
Magnesium	−4.28	

**Table 2:** Advantages and disadvantages of the HERS vs. other detection methods [21].

Detection Method	Advantages	Disadvantages
Whiff test	Low-cost, rapid	Requires reagent addition, subjective, non-quantitative, low sensitivity/specificity
Gram stain	Gold standard, excellent sensitivity/specificity	Time intensive, requires skilled personnel, laboratory facility, and reagent addition
Sialidase detection (BVBlue)	Rapid, moderate sensitivity/selectivity	Requires reagent addition, costly, non-quantitative, cannot be used by women who have recently engaged in sexual intercourse
HERS	Low-cost, rapid, quantitative, good sensitivity/selectivity	Not yet clinically validated, requires off-body analysis (not fully wearable)

## Data Availability

Data are contained within the article.

## Abbreviations

The following abbreviations are used in this manuscript:

BV	bacterial vaginosis
TMA	Trimethylamine
TMA${}^{+}$	Trimethylammonium
LOD	limit of detection
sVF	simulated vaginal fluid
cVF	commercial vaginal fluid
HERS	Home-Based Electrochemical Rapid Sensor
DI	deionized
